# Coronary Stent Infection Reimagined: A Non-surgical Victory Against a Rare Cardiac Complication

**DOI:** 10.7759/cureus.102790

**Published:** 2026-02-01

**Authors:** Gopala Krishna Medarametla, Rahul Choudhary, Surender Deora, Narendra Bordiya, Ankit Yadav

**Affiliations:** 1 Cardiology, All India Institute of Medical Sciences, Jodhpur, Jodhpur, IND

**Keywords:** bacterial infection, coronary stent, coronary stent infection, drug-eluting stent, pci (percutaneous coronary intervention), pet scans, tte: trans-thoracic echocardiography

## Abstract

Bacterial infection of a coronary stent is an exceptionally rare complication. Diagnosing such infections is often difficult due to non-specific clinical features and the limited sensitivity of early imaging studies. However, modalities like coronary angiography, cardiac CT, and PET scans can provide valuable diagnostic insights. Treatment generally involves prolonged intravenous antibiotic therapy, often in conjunction with surgical intervention when feasible. In cases where surgery carries substantial risk, conservative medical management may be considered. This report describes the case of a 36-year-old male patient who developed persistent fever shortly after coronary stent placement. The clinical course, imaging findings, and therapeutic challenges are discussed to highlight the complexities of diagnosing and managing this rare condition.

## Introduction

Coronary stent infections are extremely uncommon (incidence < 0.1%), with fewer than 50 documented cases reported globally since the widespread use of stents began [1]. Despite their rarity, they carry a significant mortality risk [2,3] and require a high degree of suspicion for timely diagnosis. The diagnosis of coronary stent infection should be among the differential diagnoses in patients with fever and a history of stent placement in the previous four weeks [4]. We report a case of coronary stent infection in a patient who developed fever after undergoing percutaneous coronary intervention (PTI) with a drug-eluting stent (DES) at a local hospital. Multimodality imaging was essential in making the diagnosis of a coronary stent infection, and the patient was successfully treated with antibiotics, without the need for surgery. 

## Case presentation

A 36-year-old man presented at our hospital with a chief complaint of fever for one week.

**Figure 1:**
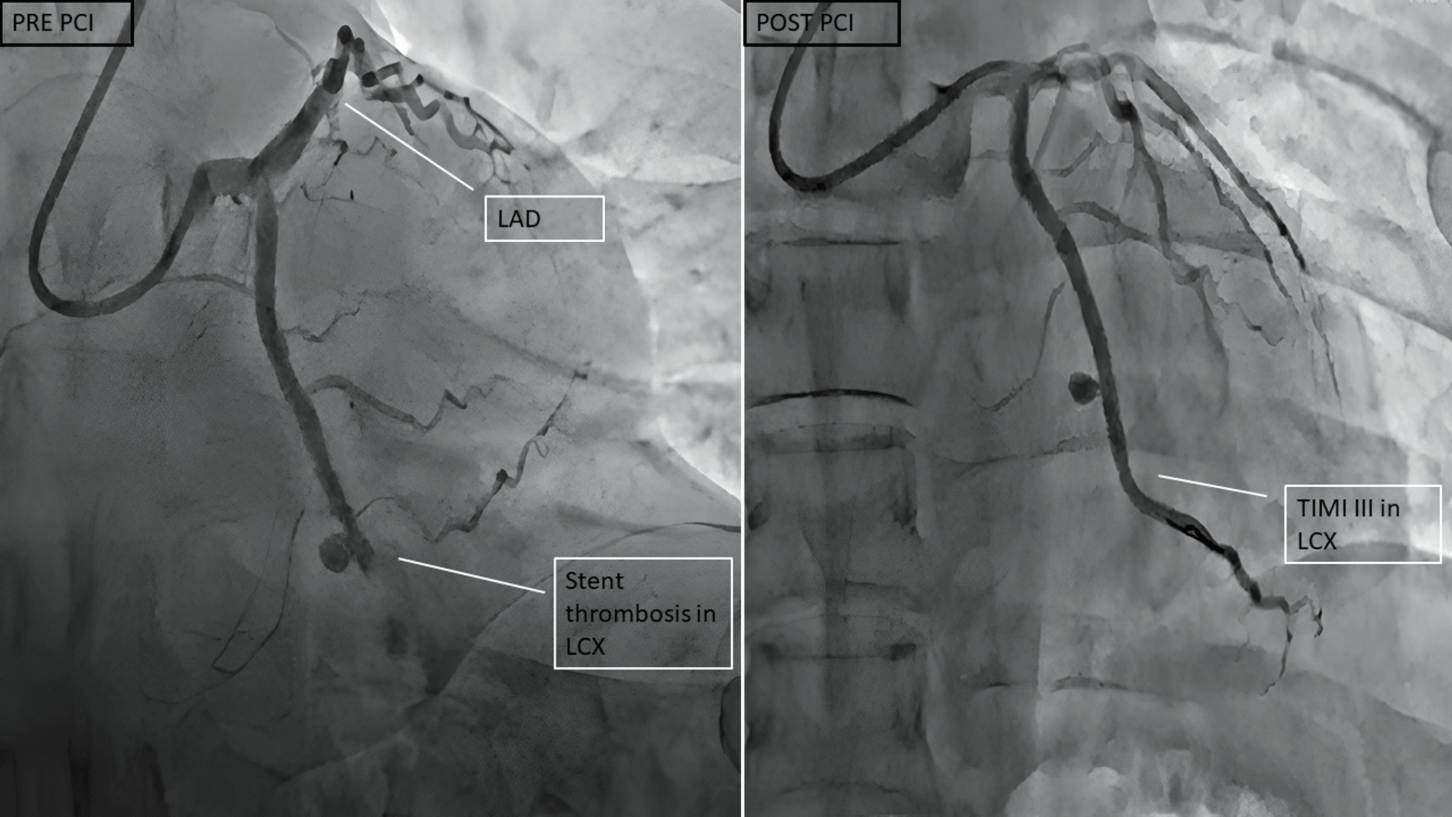
Coronary angiogram during previous admission at local hospital Pre-PCI coronary angiogram (left panel) is suggestive of aneurysm and stent thrombosis in the left circumflex vessel; Post-PCI (right panel) image shows TIMI III flow in the LCX. LAD: left anterior descending artery; LCX: left circumflex artery; PCI: percutaneous coronary intervention; TIMI: Thrombolysis in Myocardial Infarction grade flow

Three months earlier, he had suffered from an inferior wall myocardial infarction (IWMI), which was successfully treated with one DES to the left circumflex coronary artery (LCX) at a local hospital. Ten days ago, he had presented to the local hospital with a history of chest pain and was diagnosed with unstable angina. Coronary angiography was done, which was suggestive of stent thrombosis in the LCX (Figure 1). PCI was done through radial access, and stenting was done with two DES without any complications. He had a high-grade fever two days after the procedure and was managed with intravenous antibiotics at the local hospital and discharged.

**Figure 2:**
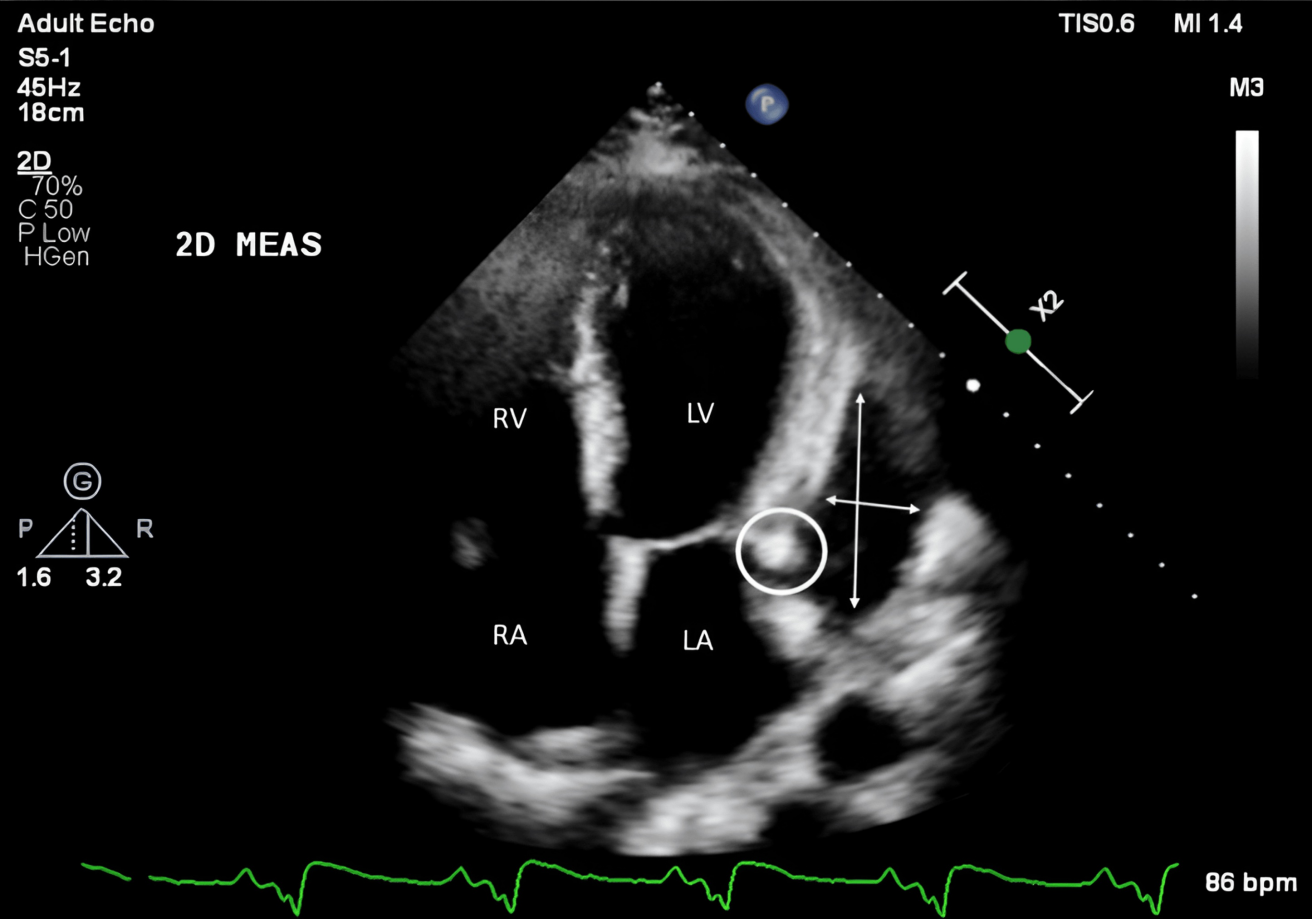
Transthoracic echocardiography suggestive of a hyperechoic structure in the left atrioventricular groove (white circle) and a localized pericardial effusion (white arrows) RA: right atria; RV: right ventricle; LA: left atria; LV: left ventricle

**Figure 3:**
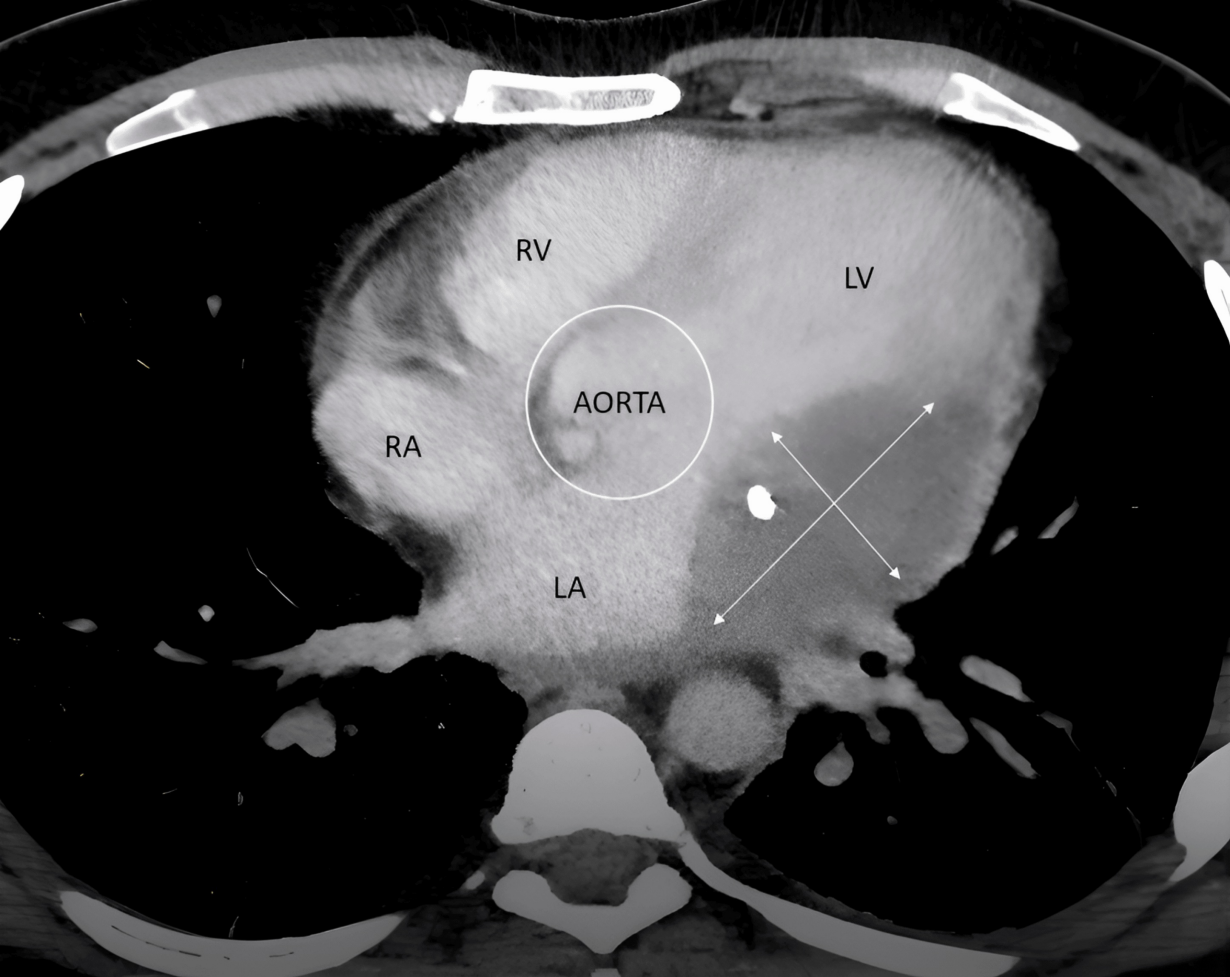
Cardiac CT suggestive of abscess in the left atrioventricular groove RA: right atrium; RV: right ventricle; LA: left atrium; LV: left ventricle

The patient was admitted to our hospital one week after the PCI at the local hospital in view of high-grade fever and started on intravenous antibiotics. Laboratory evaluation revealed elevated inflammatory markers, but the repeated blood cultures were negative. Transthoracic echocardiography revealed a suspicious echogenic lesion in the left atrioventricular groove and a localised pericardial effusion (Figure 2). Cardiac computed tomography (CT) revealed an abscess in the corresponding area, along with a lack of contrast opacification in the distal LCX stent segment, indicating potential occlusion (Figure 3).

**Figure 4:**
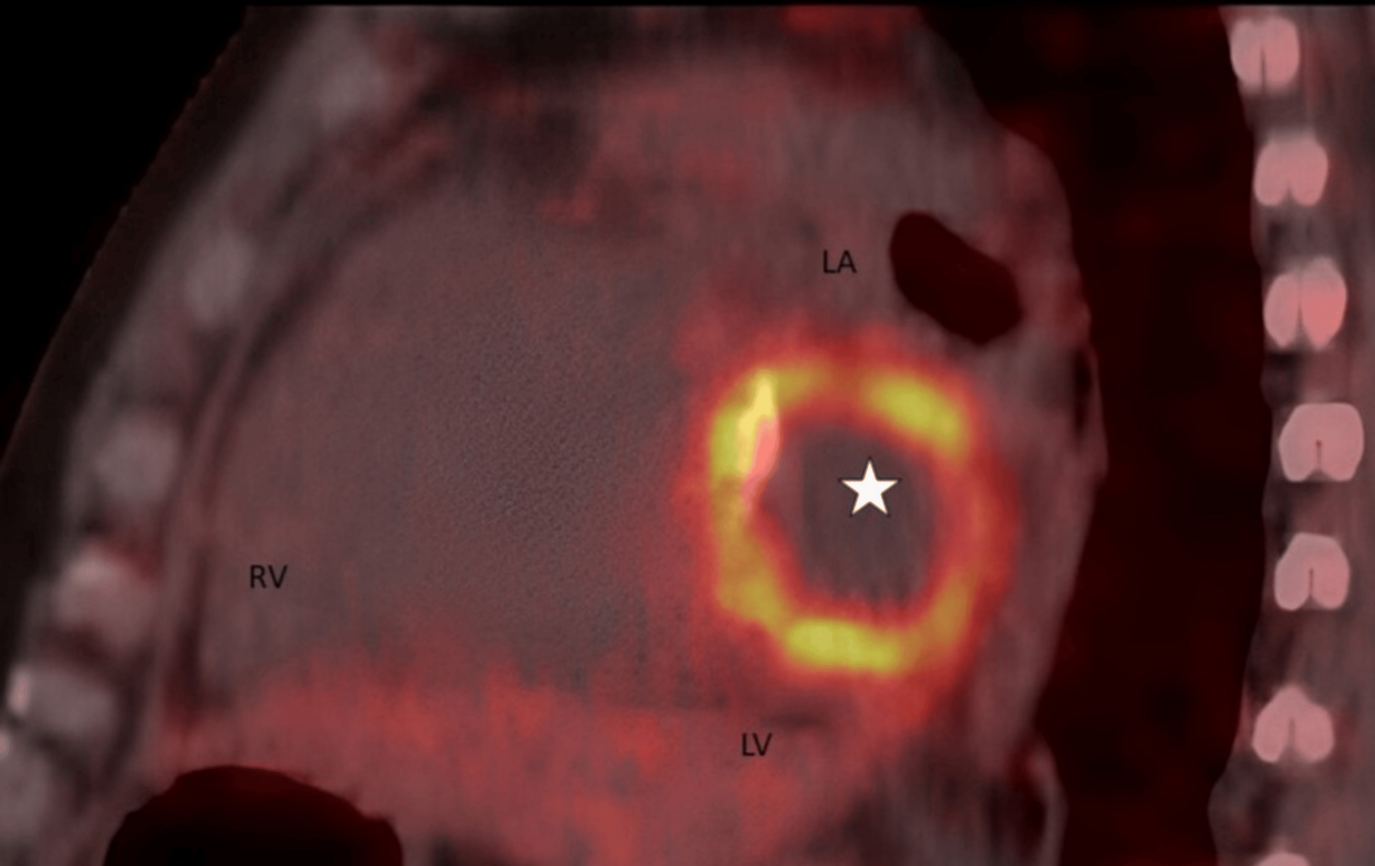
Positron emission tomography scan suggestive of increased FDG uptake along the stent in the left atrioventricular groove suggestive of coronary stent infection (star) FDG: fluorodeoxyglucose; RV: right ventricle; LA: left atrium; LV: left ventricle

A positron emission tomography (PET) scan was suggestive of localised increased metabolic activity around the stent, reinforcing the diagnosis of a coronary stent infection (Figure 4). The patient was planned for surgical intervention with abscess drainage and stent extraction, but the patient declined operative intervention in view of the risk involved.

The patient was instead managed conservatively with a six-week course of intravenous vancomycin and gentamicin in the hospital. He responded favourably to treatment, remained stable without fever spikes, and was discharged home after six weeks with a prescription for oral antibiotics, cefixime, for two weeks.

## Discussion

Coronary stent infections are an exceedingly rare complication seen after coronary artery stenting, with reported mortality rates of 40-60% [5]. It should be considered in patients developing fever following coronary stent infection in the preceding four weeks without any localisation of infection foci or bacteraemia.

Dieter proposed the diagnostic criteria for coronary stent infections [6]. A definitive diagnosis is established by directly identifying the infected stent, either during surgical exploration or at autopsy. Possible diagnosis can be made if there is presence of any three of the following: (i) coronary artery stenting in the last four weeks, (ii) complications at the site of arterial puncture site, (iii) Bacteraemia, (iv) significant fever (> 101.5 ºF) in the absence of known bacterial infection, (v) leukocytosis in the absence of known bacterial infection, (vi) acute coronary syndrome, (vii) cardiac imaging consistent with persistent inflammation [6].

Fever is the most common presentation in these patients, followed by acute coronary syndrome, stent thrombosis, and pericardial effusion [1]. The most common time of presentation is within four weeks of stenting, but presentation as late as five years has been reported [7]. DES inhibits neointimal growth, which may leave portions of the stent struts exposed, creating a potential site for bacterial adherence and subsequent infection [8]. 

Echocardiography, a widely accessible diagnostic tool, can detect vegetations, pericardial effusion, or coronary aneurysms [9]. In this case, TTE revealed an echogenic mass in the left atrioventricular groove with localised pericardial effusion, raising suspicion of stent infection. Imaging techniques such as cardiac CT, cardiac MRI, and PET scan may also aid in diagnosing stent infections when echocardiograms are inconclusive [9]. Cardiac CT was suggestive of an abscess in the left atrioventricular groove and stent thrombosis of the LCX. The PET scan was suggestive of increased FDG uptake along the stent in the left atrioventricular groove, suggestive of coronary stent infection.

Coronary stent infections can lead to serious complications such as mycotic aneurysms, pseudoaneurysms, abscesses, pericardial empyema, and purulent pericarditis [10]. In rare cases, coronary-cameral fistulas may also occur [11]. Early onset infections occur within 10 days of stenting and may respond to antibiotics alone [12]. Empirical treatment should cover *Staphylococcus aureus* and *Pseudomonas *spp, with at least four weeks of intravenous therapy recommended [12]. However, infections involving stents may persist without source removal. Late or complicated cases often require surgical stent removal, drainage, and coronary revascularisation. In the current case, the patient showed clinical improvement after a six-week course of antibiotics and continues to do well on follow-up.

## Conclusions

Coronary stent infections, though rare, should be considered in patients presenting with persistent fever following PCI. Multimodality imaging techniques are crucial for the prompt diagnosis of coronary stent infections. While surgical intervention remains the definitive treatment, early antibiotic therapy may lead to favourable outcomes in selected cases where surgery is high-risk or not feasible. In this patient, surgical management was deferred because the patient had not consented to the procedure. The patient was put on antibiotics for a six-week duration, and the condition resolved. The patient was doing well in the follow-up after six months and remained afebrile. 

## References

[1] Reddy KV, Sanzgiri P, Thanki F, Suratkal V (2019). Coronary stent infection: Interesting cases with varied presentation. J Cardiol Cases.

[2] Buono A, Maloberti A, Bossi IM (2019). Mycotic coronary aneurysms. J Cardiovasc Med (Hagerstown).

[3] Baddour LM, Bettmann MA, Bolger AF (2003). Nonvalvular cardiovascular device-related infections. Circulation.

[4] Bosman WM, Borger van der Burg BL, Schuttevaer HM, Thoma S, Hedeman Joosten PP (2014). Infections of intravascular bare metal stents: a case report and review of literature. Eur J Vasc Endovasc Surg.

[5] Kaufmann BA, Kaiser C, Pfisterer ME, Bonetti PO (2005). Coronary stent infection: a rare but severe complication of percutaneous coronary intervention. Swiss Med Wkly.

[6] Dieter RS (2000). Coronary artery stent infection. Clin Cardiol.

[7] Doost A, Ihdayhid AR, Lambert J, Erickson M (2021). Very late coronary stent infection and abscess following Staphylococcus aureus bacteremia. CASE (Phila).

[8] Pisani A, Braham W, Borghese O (2021). Coronary stent infection: Are patients amenable to surgical treatment? A systematic review and narrative synthesis. Int J Cardiol.

[9] Kumar SS, Suresh S, Iliyas M, Vijay J, Pillai V (2024). A case report of left circumflex stent infection and mycotic aneurysm: a rare but life-threatening complication of percutaneous coronary intervention. Egypt Heart J.

[10] Roubelakis A, Rawlins J, Baliulis G, Olsen S, Corbett S, Kaarne M, Curzen N (2015). Coronary artery rupture caused by stent infection: a rare complication. Circulation.

[11] Sangolkar R, Ketana VR, Sastry BK (2018). Coronary artery stent infection presenting as coronary cameral fistula: a case report. Eur Heart J Case Rep.

[12] Elieson M, Mixon T, Carpenter J (2012). Coronary stent infections: a case report and literature review. Tex Heart Inst J.

